# Cholelithiasis in a goat associated with chronic fascioliosis in Bangladesh: A case report and review of literatures

**DOI:** 10.1002/vms3.1476

**Published:** 2024-05-20

**Authors:** Md. Haydar Ali, Sharmin Shahid Labony, Md. Shahadat Hossain, Md. Mahmudul Alam, Md. Abu Hadi Noor Ali Khan, Md. Abdul Alim, Anisuzzaman

**Affiliations:** ^1^ Department of Parasitology Bangladesh Agricultural University Mymensingh Bangladesh; ^2^ Department of Pathology and Parasitology Hajee Mohammad Danesh Science and Technology University Mymensingh Bangladesh; ^3^ Department of Surgery and Obstetrics Bangladesh Agricultural University Mymensingh Bangladesh; ^4^ Department of Pathology Bangladesh Agricultural University Mymensingh Bangladesh

**Keywords:** Black Bengal Goats, *Fasciola gigantica*, gallstone

## Abstract

The objective of the present study was to describe a very rare case of gallstone (cholelithiasis) in a goat associated with chronic fasciolosis. During a routine slaughterhouse‐based survey, a two‐and‐half‐year‐old female Black Bengal Goat was found to be affected with severe chronic fascioliosis characterized by the massive damage in the liver. Through systemic dissection of liver, we isolated 94 adult *Fasciola* spp., and by PCR, we confirmed the fluke as *Fasciola gigantica*. The gallbladder of the goat was oedematous. On opening the gallbladder, we recovered 255 stones of variable sizes. Stones were whitish in colour and friable, and some of the fragile stones were attached to the wall of the gallbladder. To the authors’ knowledge, this is the first report of the cholelithiasis in a goat associated with *F. gigantica*.

## Graphical abstract

❖ During a routine slaughterhouse survey, a two and a half‐year‐old female Black Bengal Goat was found to be affected with severe chronic fascioliosis characterized by massive damages in the liver.

❖ We isolated 94 adult *Fasciola* parasites from the affected liver which were confirmed as *Fasciola gigantica* by PCR.

❖ We recovered 255 friable whitish stones of variable sizes some of which were attached to the wall of the gallbladder that induced adenomatous cholecystitis.

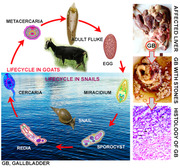

## 1 INTRODUCTION

Fascioliosis is a zoonotic disease caused by *Fasciola gigantica* and *Fasciola hepatica* (Trematoda: Fasciolidae), which are commonly known as liver flukes. In Bangladesh, *F. gigantica*, but not *F. hepatica*, is highly prevalent (Rahman et al., 2017; Yasin et al., 2018). It is a food‐ and waterborne parasitic disease. Gastropod snails, especially *Lymnaea auricularia* and *Lymnaea luteola* act as intermediate hosts of *F. gigantica* in Bangladesh, in which the fluke develops into several asexual stages and eventually the cercarial stages are released. Metacercaria usually develops on grass blade, and final hosts, especially cattle, buffaloes, sheep and goats, become infected during grazing (John et al., 2019).

In goats, liver flukes cause severe liver damages leading to liver cirrhosis, cholangitis, and thickening of the bile ducts; thus, they cause hepatic dysfunction, hypoproteinemia, oedema, bottle jaw, stunted growth, weight loss, production loss and ultimately badly affected animals may die (Elelu & Eisler, 2018). Fascioliosis is associated with huge economic losses and WHO has listed the disease to the list of neglected tropical diseases, and about 17 million people are infected and 180 million people are at risk (Caravedo et al., 2021).

Here, we, for the first time, present a case of severe cholelithiasis in a Black Bengal Goat (BBG) associated with *F. gigantica* infection.

## 2 CASE SUMMARY

2

**FIGURE 1:**
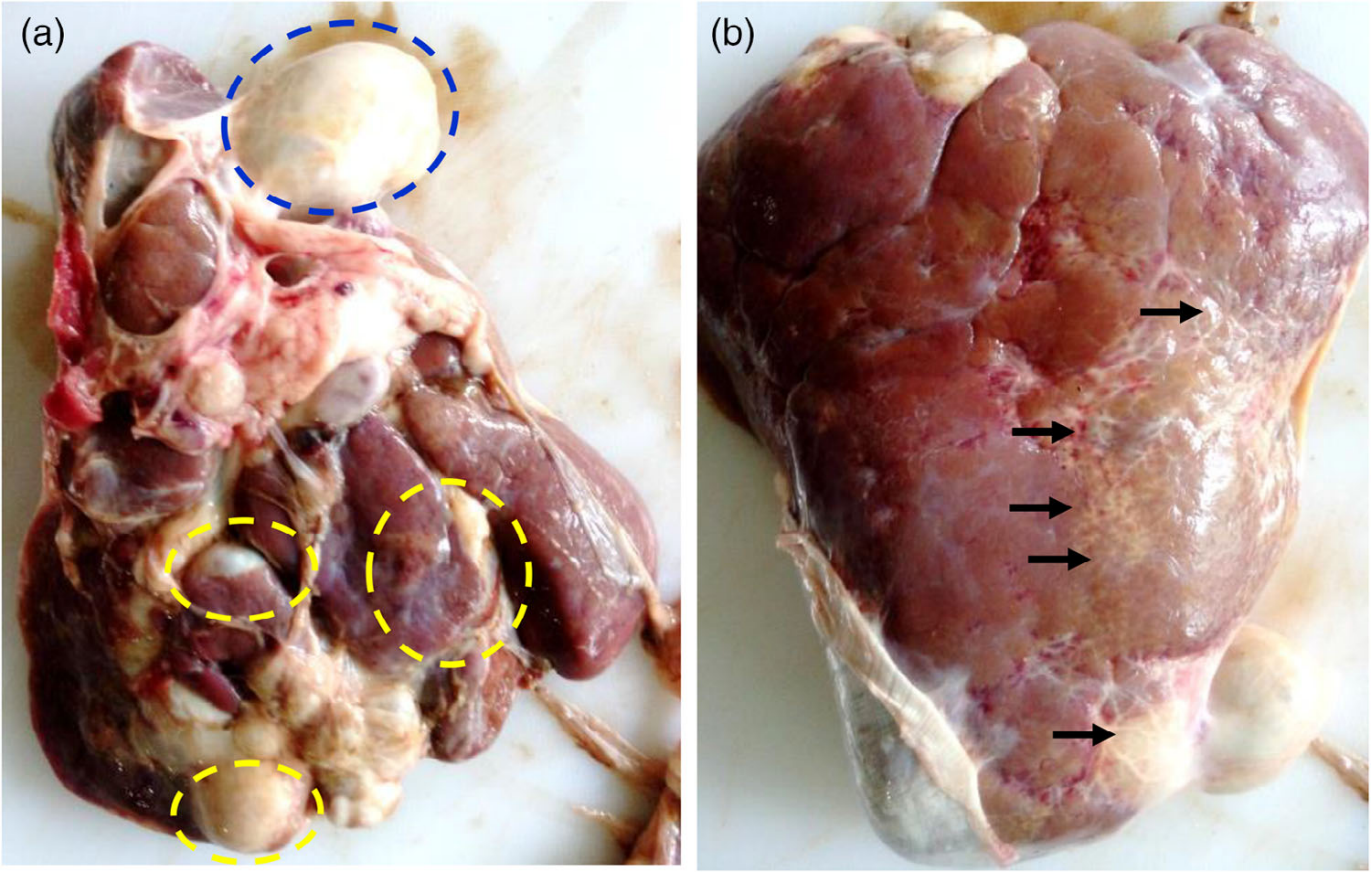
**The affected** **liver of the goat**. (a) visceral view. Blue circle, gallbladder; yellow circle, cyst‐like structure in the liver that contained flukes and tarry coloured fetid materials. (b) parietal surface of the liver. Arrows indicate grossly white patches of fibrosis.

**FIGURE 2:**
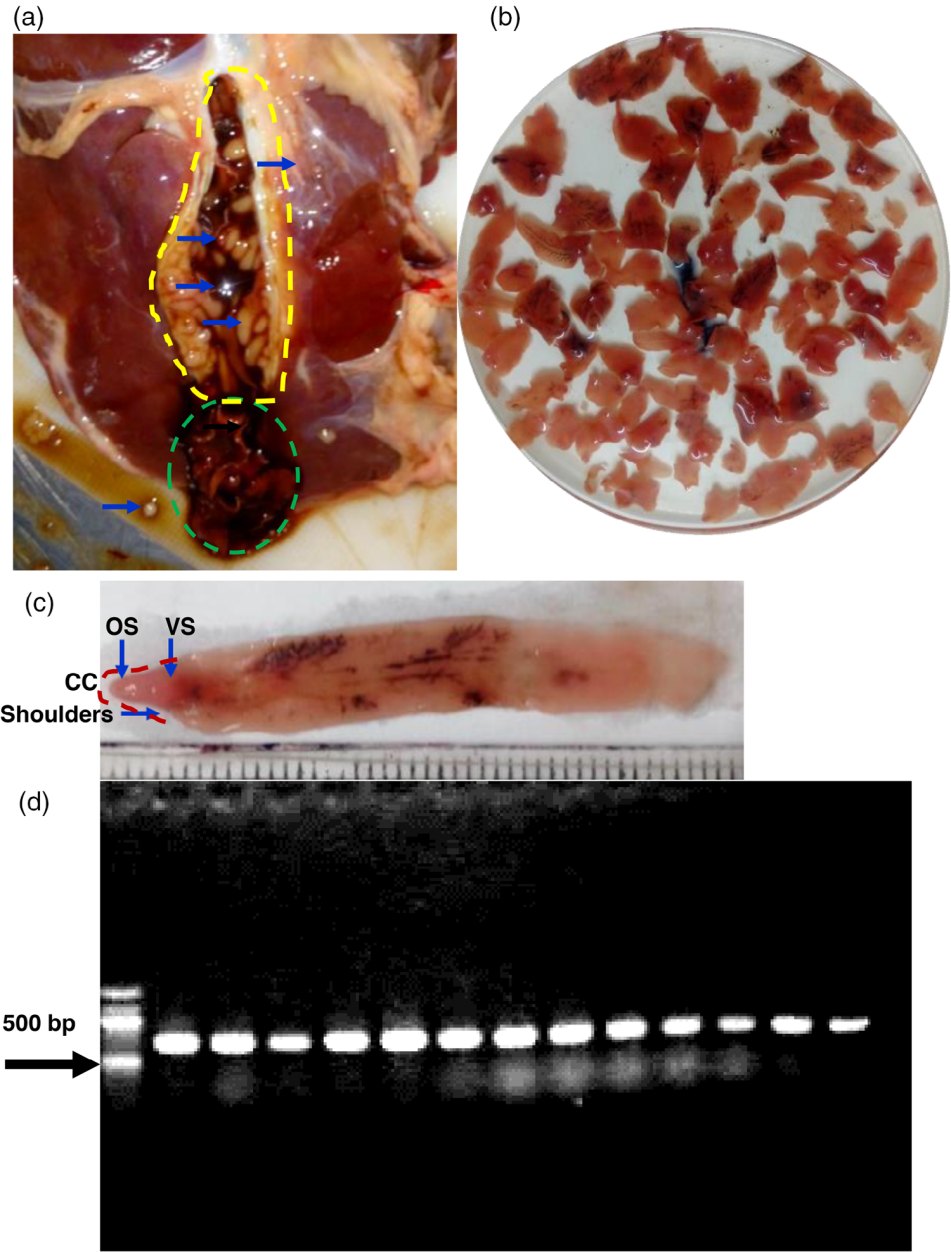
**Identification of *Fasciola gigantica***. (a) cut surface of the affected liver showing aggressed adult fluke. (b) collected adult flukes (c) showing different structure of an adult fluke. CC., cephalic cone; OS, oral sucker; VS, ventral sucker. (d) internal transcribed spacer (ITS1)‐based confirmation of species of *Fasciola gigantica*.

During our routine slaughterhouse survey, a two‐and‐half‐year‐old female BBG was found to be affected with chronic fascioliosis characterized by the severe damages in the liver. We brought the liver and gallbladder to the Diagnostic Parasitology Laboratory, and conducted systemic examination of the affected liver. Upon gross examination, we observed that the entire liver was swollen and oedematous. There were whitish patches of fibrous mass deposition both on the visceral and parietal surface of the liver. In the visceral surface, several cysts‐like growths were evident (Figure 1a,b). We placed a tunicate at the neck of the gallbladder on the cystic duct and separated the gallbladder. The liver was dissected along the course of the bile ducts. Moreover, we opened the gallbladder, and parasites were collected. While dissecting, fetid odoured, tarry coloured, viscous fluid oozes out along with the crawling of adult *F. gigantica*, indicating that the parasites themselves produced such cystic or pocket‐like structures due to dilatation of bile ducts in the liver, where they lived in (Figure 2a).

Parasites were gently removed from the lumen of the dissected bile ducts and washed with phosphate‐buffered saline (PBS). For tentative identification, morphological and morphometrical analyses of the flukes were done. All the flukes were elongated (4.1–7.2 cm long × 0.9–1.2 cm wide) and dorsoventrally flattened, unsegmented, distome (with prominent oral sucker and ventral sucker) with prominent shoulders; thus, they formed a distinct cephalic cone. The ventral sucker of the fluke was at the level of shoulders (Figure 2b,c). We isolated a total of 94 adult flukes from the gallbladder and bile ducts. Then we prepared permanent slide by staining the fluke with Semichon's carmine as described previously (Soulsby, 1982), and microscopic study revealed that the intestinal ceca, ovary and testes of the flukes were heavily branched, conforming to the morphological features of *F. gigantica*. Then we cut only the cephalic cone of each fluke and extracted genomic DNA (gDNA) using QIAamp DNA Mini Kit following the manufacturer's instructions. The concentration of the extracted gDNA was measured using a nanodrop spectrophotometer. To confirm the species, internal transcribed spacer gene was amplified using species‐specific primers as described previously (Mohanta et al., 2014). The PCR thermal cycle consisted of one cycle of 2 min at 94°C, 35 cycles of 1 min at 93°C, 1 min at 55°C and 1 min at 72°C each, followed by 2 min at 72°C, and final cooling at 4°C. Then, the PCR products were subjected to electrophoresis in a 1.5% agarose gel. By PCR and subsequent electrophoresis, we got desired bands at the expected level in all flukes used, confirming the fluke as *F*. *gigantica* (Figure 2d).

**FIGURE 3:**
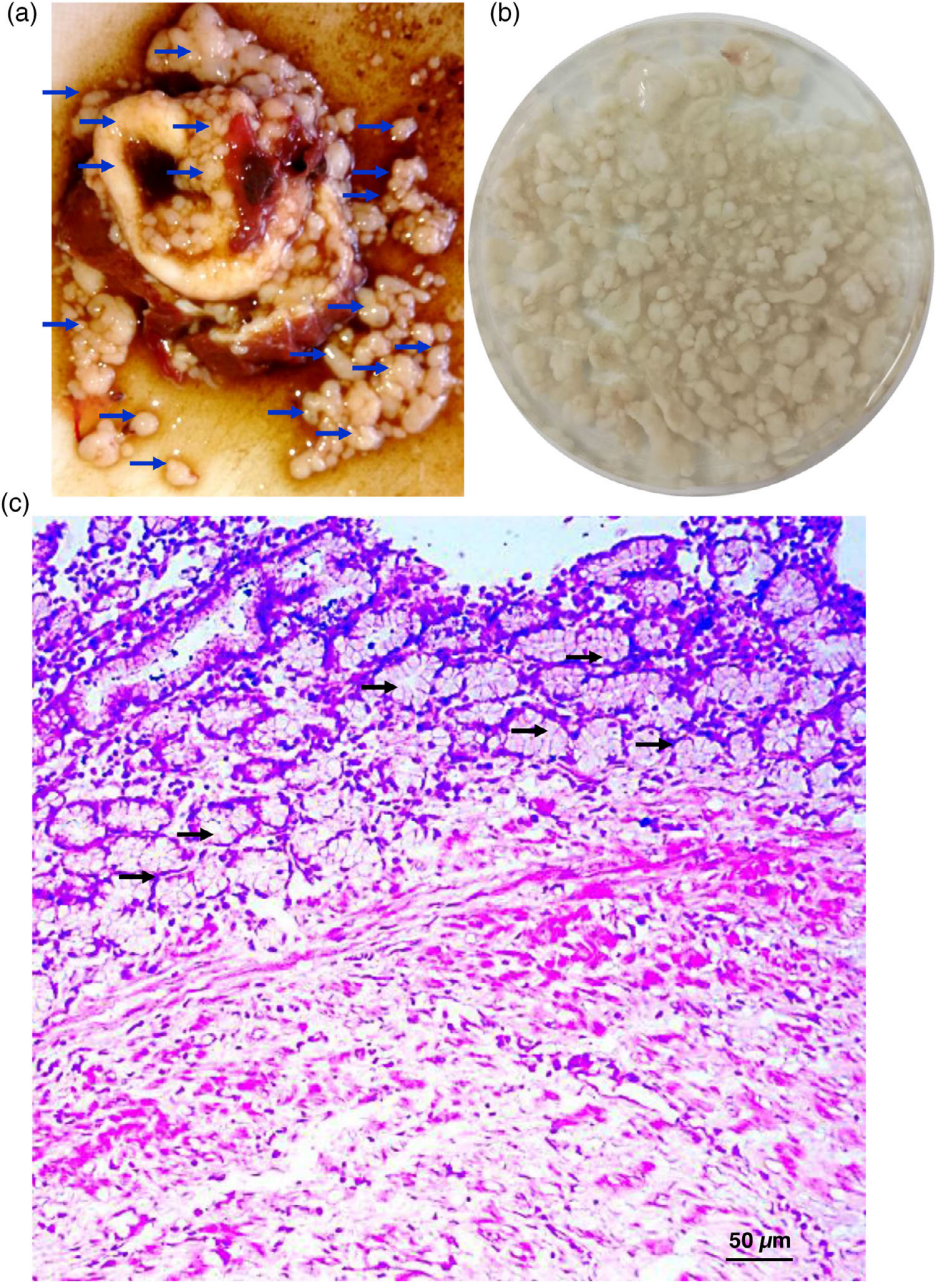
**Cholelithiasis in the affected liver of the goat**. (a) exposed gallbladder containing stones of variable sizes. Arrows indicate stones (b) collected stones. (c) histopathological section of the wall of the affected gallbladder showing adenomatous cholangitis.

Although we opened the gallbladder, we found numerous stones of variable size. We collected the stones and gently washed them with PBS and counted. We could detect ∼255 stones of variable sizes mostly with irregular shape; however, some stones were circumscribed and shiny (Figure 3a,b). Some stones were friable but some were very hard; felt upon digital pressure. In addition to gallbladder, few stones were also detected in common bile ducts and in intrahepatic bile ducts. Wall of the gallbladder was very thick, and some friable whitish mass with irregular surface was also found attached to the internal surface of the gallbladder. Bile found within the gallbladder was very viscous, turbid and tarry colour with fetid odour. Then we took a piece of tissue (∼5 mm^2^) and preserved in a fixative containing absolute alcohol and glacial acetic acid (3:1) for 48 h under gentle shaking and washed extensively with PBS. The tissue was then embedded in paraffin, and thin (5 µm) sections were made and stained with haematoxylin and eosin stain. Microscopic examination of the sections revealed thickening of the wall of the gallbladder. There was a huge proliferation of the glandular structures in the mucous layer of the gallbladder, suggesting adenomatous cholecystitis. Proliferation of fibrous connective tissues was also evident. Erosion in the mucous layer and distortion of normal mucosal lining of the gallbladder were detected (Figure 3c). Cheema and Hooshmand‐Rad (1985) reported adenomatous cholecystitis in goats due to adult *F. gigantica* infection by giving experimental infections with the fluke.

In general, gallstones or cholelithiasis in goats is a rare event. In a study in Iran, only 1.2% goats (5 out of 411 goats) were found to be suffered from cholelithiasis; however, none of the cases were associated with fascioliosis, rather the cases of cholelithiasis were thought to be linked with bacterial infections (Raoofi et al., 2012). Another study reported periductal concentric fibrosis and cast‐like biliary microliths in three goats, which were speculated to be due to dietary origin (Collett & Spickett, 1989). However, choleliths had also been reported in cattle, which was associated with *F. gigantica* (Javaregowda & Rani, 2017). In a clinical investigation based on sheep liver examination (*n* = 254), the scientists isolated *F. hepatica*, *Dicrocoelium dendriticum*, and hydatid cysts and detected biliary calculi, either pigmented or cholesterol stones in 40 livers (Katsoulos et al., 2011). Impacts of fascioliosis on the development of gallstone have been evaluated by giving experimental infection with *F. hepatica* in Wistar rats and the researcher found that the flukes can induce gallstone, and the relative risk of development of gallstone was strongly associated with the number of flukes per rat. From their study, they speculated that fascioliosis may cause gallstone in humans in the endemic areas (Valero et al., 2003). In fact, in humans, fascioliosis has been found to be linked with gallstone in several countries (Yilmaz et al., 2004). A meta‐analysis showed that liver fluke infections caused by *Clonorchis sinensis*, *Fasciola* spp. and *Opisthorchis* spp. are significantly associated with cholangitis, cholecystitis, cholelithiasis, hepatocellular carcinoma and cholangiocarcinoma (Xia et al., 2015). However, up to now, no cases of fascioliosis‐linked cholelithiasis had been detected in goats.

## AUTHOR CONTRIBUTIONS

Md. Haydar Ali performed sample collection, data curation, formal analysis, investigation, methodology, data validation, writing—original draft; Sharmin Shahid Labony carried out methodology, writing – original draft, writing – review and editing; Md. Shahadat Hossain did data visualization, writing – original draft, writing – review and editing; Md. Mahmudul Alam performed software analysis, data visualization; Md. Abu Hadi Noor Ali Khan did conceptualization, resources support, writing – review and editing; Md. Abdul Alim conceptualization, resources support, data validation; Anisuszzaman did conceptualization, project administration, supervision, writing – review and editing.

## CONFLICT OF INTEREST STATEMENT

The author(s) declared no potential conflicts of interest with respect to the research, authorship and/or publication of this article.

## FUNDING INFORMATION

The author(s) received no financial support for the research, authorship and/or publication of this article.

### ETHICS STATEMENT

No ethical issues were involved for this study.

### PEER REVIEW

The peer review history for this article is available at https://publons.com/publon/10.1002/vms3.1476

## Data Availability

The datasets used and/or analysed during the current study are available from the corresponding author on reasonable request.
